# Transcranial ultrasound analysis of cerebral blood flow during induced hypertension in acute ischemic stroke - a case series

**DOI:** 10.1186/2036-7902-5-4

**Published:** 2013-04-08

**Authors:** Jonathan List, Jens E Röhl, Florian Doepp, José M Valdueza, Stephan J Schreiber

**Affiliations:** 1 Department of Neurology, Charité - Universitätsmedizin Berlin, Charitéplatz 1, Berlin 10117, Germany; 2 Center for Stroke Research Berlin, Charité - Universitätsmedizin Berlin, Berlin 10117, Germany; 3 Department of Neurology, St. Josefs Krankenhaus, Potsdam 14471, Germany; 4 Department of Neurology, Segeberger Kliniken, Bad Segeberg 23795, Germany

**Keywords:** Transcranial ultrasound, Acute stroke, Cerebral autoregulation, Induced hypertension

## Abstract

**Background:**

Current recommendations of stroke treatment favour a moderately elevated blood pressure in the acute phase, based on the concept of an improved cerebral perfusion. Here, cerebral blood flow was assessed in a case series of patients with acute hemodynamic stroke by means of transcranial colour-coded sonography (TCCS) to study the effects of pharmacologically induced hypertension.

**Findings:**

We investigated six patients with acute hemodynamic stroke and blood pressure-dependent clinical fluctuation of neurological symptoms. TCCS was performed during the initiation phase of catecholamine-induced controlled hypertension. A blood pressure-dependent increase of flow velocity in the ipsilesional middle and the posterior cerebral artery was found in all patients (mean increase 0.80% and 0.65% per mmHg, respectively).

**Conclusions:**

Catecholamine-induced hypertension in severe hemodynamic stroke leads to an ultrasound-detectable rise of cerebral blood flow. This finding gives ‘proof-of-principle’ evidence, supporting active blood pressure management in this selected group of stroke patients. Outcome-related questions of target blood pressure, treatment duration or applicability to other forms of stroke, however, remain to be studied. In this, transcranial ultrasound may be a valuable tool for patient selection and subsequent bedside monitoring.

## Findings

### Introduction and background

Cerebral autoregulation refers to the ability to keep cerebral blood flow (CBF) stable, independent of systemic blood pressure (BP) [1]. In ischemic stroke, a failure of autoregulation is assumed, leading to current treatment guidelines of permissive hypertension in the hyperacute phase [2]. However, the actual effect of BP changes on CBF in acute stroke patients is poorly investigated and never routinely assessed.

We report an observational case series, analysing the effects of pharmacologically induced hypertension on CBF in a subgroup of stroke patients with occlusive disease of the internal carotid artery (ICA) by means of transcranial colour-coded sonography (TCCS).

### Patients and methods

Acute stroke patients were prospectively included if they fulfilled the following criteria: (1) proximal occlusion or high-grade stenosis of the ICA (>80%, ECST criteria [3]), (2) ultrasound-determined intracranial collateral flow activation (retrograde ipsilesional flow in the A1 segment of the anterior cerebral artery (ACA), activated posterior communicating artery, retrograde ophthalmic artery flow or signs of leptomeningeal collateral activation, i.e. increased flow velocity in the distal ACA or posterior cerebral artery (PCA)), (3) BP-related worsening of the neurological deficits (NIH-SS ≥1), (4) presumed haemodynamic cause because of marked poststenotic flow patterns in the ipsilesional middle cerebral artery (MCA) and (5) subsequent continuous intra-arterial BP monitoring and need for catecholamine intervention to achieve or keep a BP target of systolic 160 to 190 mmHg.

All patients had already completed extracranial colour-coded sonography (ECCS) and TCCS studies (Toshiba Powervision 6000, Tokyo, Japan) including detailed intracranial collateral flow assessment during the routine process of acute stroke unit admission. Without interference to clinical decisions and procedures, serial measurements of non-angle-corrected blood flow velocities were performed in the ipsilateral M1-MCA and P2-PCA segments during initiation of continuous catecholamine infusion. Datasets of maximal flow velocities and corresponding systolic BP values were noted, following a stepwise increase of catecholamine dosage and a stabilisation phase of 2 to 3 min. All patients gave informed consent; the study was approved by the local ethics committee.

### Results

**Figure 1:**
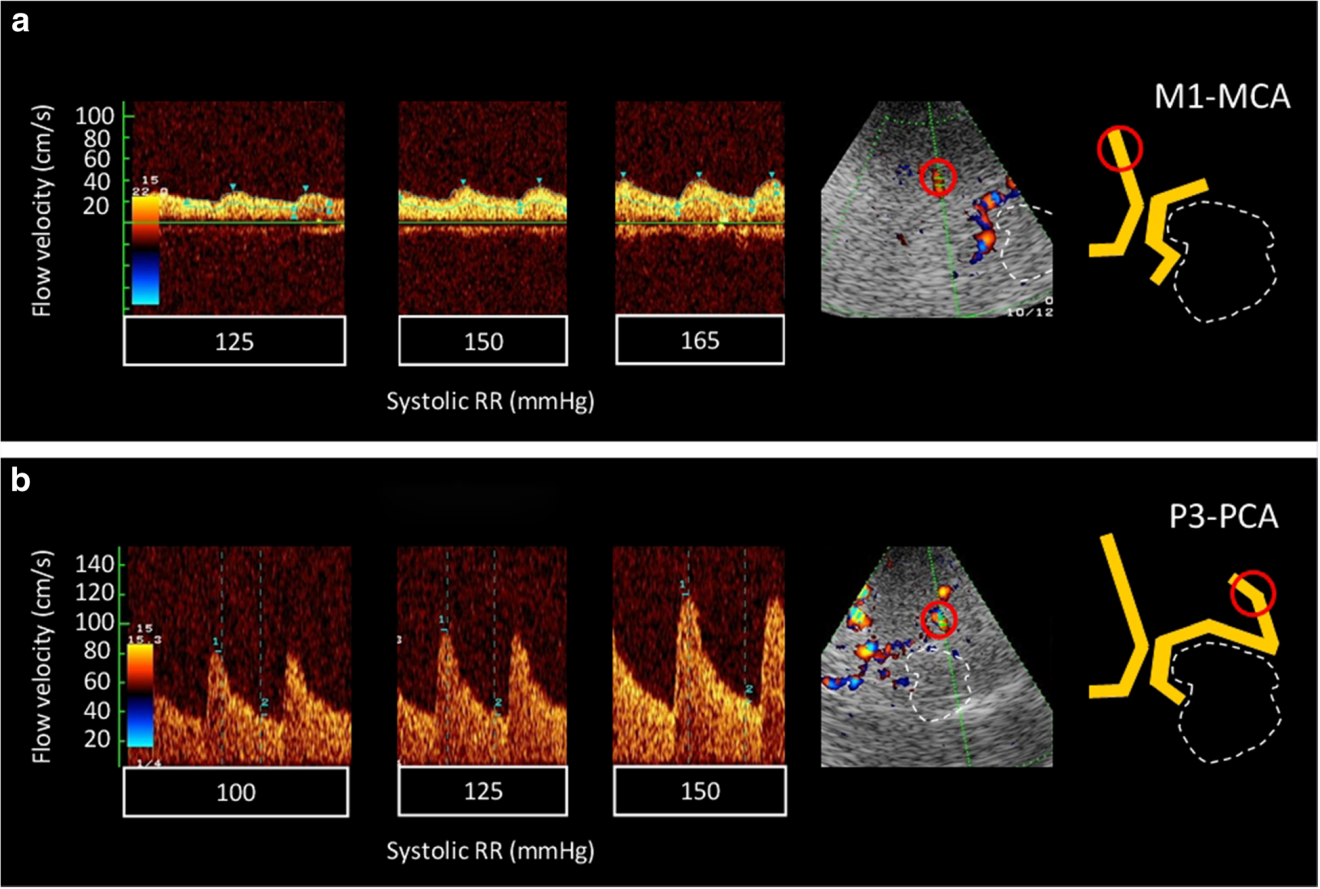
**Example of intracranial blood pressure-dependent flow velocity changes. **(**a**) M1-MCA segment (**b**) P3-PCA segment. Placement of the Doppler sample volume is marked by red circles in the original colour-mode image and in a corresponding schematic drawing (right). The white dotted lines indicate the location of the midbrain. The individual systolic flow velocities at the above blood pressure values were for the MCA (**a**): 125 mmHg - 28/21 cm/s; 150 mmHg - 39/26 cm/s; 165 mmHg - 45/26 cm/s and for the PCA (**b**): 100 mmHg - 81/31 cm/s; 125 mmHg - 96/38 cm/s; 150 mmHg - 119/46 cm/s.

Six patients (five male, one female; 58 to 81 years) were included (Table 1). Cerebral magnetic resonance imaging (MRI) revealed acute MCA-ACA watershed infarctions or MCA inner borderzone infarction. ECCS demonstrated ipsilateral high-grade ICA stenosis (>90%) in two and acute ICA occlusion in four of our patients. TCCS revealed impaired collateralisation with poststenotic ipsilateral MCA flow (delayed systolic flow rise, reduced pulsatility and flow velocities) in all subjects. The clinical indication for norepinephrine treatment in all our patients was set within the first 24 h after stroke onset. Stepwise dosage increase of continuous catecholamine infusion led to BP rises ranging from 25 to 100 mmHg. A blood pressure-dependent increase of the peak systolic flow velocity was observed in the M1-MCA (mean increase of 0.80% per mmHg) as well as in the P2-PCA of the affected side (mean increase of 0.65% per mmHg) (Figure 1a,b, Table 1).

**Table 1:** Patient characteristics and ultrasound results

**No.**	**Age, gender**	**Side**	**NIH-SS baseline/during IHT**	**Collateral pathways**	**MCA (affected side)**			**PCA (affected side)**		
					**PSV**_**baseline**_	**PSV**_**IHT**_	**PSV**_**increase**_	**PSV**_**baseline**_	**PSV**_**IHT**_	**PSV**_**increase**_
					**(BP)**	**(BP)**	(**% per mmHg)**	**(BP)**	**(BP)**	(**% per mmHg)**
1	66, M	Left, O	2/0	ROA, PCOA, LP	21 cm/s	29 cm/s	0.38	81 cm/s	160 cm/s	0.98
					(100 mmHg)	(200 mmHg)		(100 mmHg)	(200 mmHg)	
2	81, M	Right, S	2/2	RACA, ROA, LP	51 cm/s	61 cm/s	0.78	83 cm/s	102 cm/s	0.92
					(105 mmHg)	(130 mmHg)		(105 mmHg)	(130 mmHg)	
3	76, M	Right, S	5/3	RACA	70 cm/s	85 cm/s	0.71	NA	NA	NA
					(185 mmHg)	(215 mmHg)				
4	71, M	Right, O	2/2	ROA, RACA	29 cm/s	37 cm/s	0.61	66 cm/s	82 cm/s	0.37
					(162 mmHg)	(207 mmHg)		(162 mmHg)	(227 mmHg)	
5	71, M	Right, O	3/NA	ROA, RACA	24 cm/s	49 cm/s	1.45	NA	NA	NA
					(120 mmHg)	(190 mmHg)				
6	58, F	Right, O	3/3	ROA, RACA	33 cm/s	45 cm/s	0.89	199 cm/s	227 cm/s	0.34
					(124 mmHg)	(165 mmHg)		(124 mmHg)	(165 mmHg)	

Abbreviations: O, occlusion; S, stenosis; NIH-SS, NIH stroke scale; ROA, retrograde ophthalmic artery; RACA, retrograde A1-anterior cerebral artery; LP, leptomeningeal via PCA; PCOA, activated posterior communicating artery; PSV, peak systolic flow velocity; NA, not assessed; IHT, induced hypertension; BP, blood pressure; No., patient number; MCA, middle cerebral artery; PCA, posterior cerebral artery.

Patients received catecholamine treatment for 1 to 14 days. Constant achievement of BP targets initially led to an instant clinical stabilisation in all patients. Patients 1 and 3 improved as indicated by NIH-SS. Patient 1 was subsequently treated by extra-intracranial bypass operation, as aphasia and a hemiparesis repeatedly occurred during attempts of catecholamine withdrawal. Patients 2 and 3 received carotid endarterectomy and cartotid stenting, respectively. Patient 4 stabilised with spontaneous hypertensive blood pressure values after 24 h. Patient 5 developed fatal malignant MCA infarction on the second day. Patient 6 remained stable under catecholamine medication; however, she worsened from mild paresis to hemiplegia during attempts of slow catecholamine withdrawal after 14 days of continuous treatment.

### Discussion

Current acute stroke treatment concepts comprise blood pressure monitoring and active management, based on the hypothesis of blood pressure-related improved perfusion of recoverable peri-infarct brain tissue [4]. However, confirming *in vivo* studies are rare, and it remains unclear who among the patients should actively be treated and who may not need to be treated - for how long and by which means (i.e. volume substitution alone or catecholamine-induced hypertension); BP should actively be modified. As a proof-of-concept and functional evidence for cerebral autoregulation failure, we could demonstrate that an active BP increase leads indeed to an ipsilesional increase of TCCS-detected flow velocities, i.e. to a clear treatment-induced improvement of cerebral blood flow. The mere observation of an epiphenomenon, i.e. a catecholamine-induced proximal vasoconstriction which mimics increased CBF, seems unlikely, as previous studies reported unchanged flow velocities during norepinephrine challenge [5]. Interestingly, induced hypertension did improve not only ipsilateral MCA but also ipsilateral PCA flow as an indirect sign of leptomeningeal collateral activation, giving additional insight into the pathophysiology of compensatory mechanisms.

Similar confirmatory effects of hypertension-induced improvement of cerebral perfusion have been reported in MRI-determined perfusion studies, but so far, only in the form of case reports [6,7]. Rordorf et al. selected patients for catecholamine treatment by evaluating the clinical response to a 30-min catecholamine challenge. Seven of 13 patients had a positive response and were subsequently treated for 1 to 6 days. All seven improved [8].

In contrast to MRI or CT, ultrasound is an ideal tool for continuous, repetitive or fast bedside analysis as intracranial blood flow velocity changes are a good relative estimate of cerebral blood flow alteration. Schwarz et al. analysed bilateral MCA blood flow velocities of 19 patients with large or total MCA infarctions [9]. They observed ipsilateral hyperemia and a strong ipsilateral flow velocity to blood pressure relation (1.4% rise per mmHg compared to 0.4% per mmHg contralateral). However, all their patients were sedated and mechanically ventilated on the intensive care unit. Despite the clearly seen impaired cerebral autoregulation, the expectable benefit of induced hypertension to a total MCA infarction has to be questioned.

Our inclusion criteria led to a preselection of mildly affected patients with low-flow haemodynamic infarction, because of their impaired collateral function being particularly vulnerable to secondary worsening. Corresponding to this concept, the three patients with finally successful interventions and subsequent improvement of ipsilesional cerebral blood flow did well, while the two without this option showed an unfavourable outcome. Our patient with spontaneous stabilisation might be an example of a gradual collateral adaptation.

### Limitations

Due to our study’s small sample size, clinical conclusions are limited. Our data suggest but do not prove that induced hypertension might be helpful for a short time period in the particular selected group of patients with low-flow haemodynamic infarction, but patients might still need further vascular intervention for final clinical stabilisation. Is there a clinical or haemodynamic constellation which helps to decide whether a patient will spontaneously stabilise or rather be sent for an early vascular intervention? Would the observed effects also be detectable in patients with embolic or lacunar stroke? Who among the patients are particularly benefitting from a therapeutic intervention? If applied, which target BP (systolic or mean) would be optimal for cerebral perfusion, and does this relevantly increase the risk of, e.g. a hypertension-induced cerebral haemorrhage? Which ultrasound technique should be used: a handheld Duplex, prone to, e.g. movement artefacts or head-frame-fixed transcranial Doppler probes? Is the peak systolic flow velocity sufficient, or are other parameters like mean flow velocity or pulsatility more important?

Further studies with larger patient cohorts, e.g. analysing flow response patterns in the above-discussed groups, and evaluating the indivdual extent of cerebral autoregulation failure would be required to answer some of the above aspects. To our opinion, transcranial ultrasound with its high temporal resolution and non-invasiveness is here particularly promising to become a tool for patient selection and individual tailoring, e.g. of treatment duration.

### Conclusions

In this case series, we could demonstrate that catecholamine-induced hypertension in severe haemodynamic stroke leads to an ultrasound-detectable rise of cerebral blood flow. This finding gives ‘proof-of-principle’ evidence, supporting active blood pressure management in this subgroup of acute stroke patients. Our results advocate using transcranial ultrasound to further analyse the relationship of BP-related cerebral blood flow in acute ischemic stroke and herewith to search for better and more individually tailored treatment concepts.

## Abbreviations

ACA: anterior cerebral artery; BP: blood pressure; CBF: cerebral blood flow; ECSS: extracranial colour-coded sonography; ICA: internal carotid artery; MCA: middle cerebral artery; PCA: posterior cerebral artery; TCCS: transcranial colour-coded sonography.

## Competing interests

The authors declare that they have no competing interests.

## Authors’ contributions

STS and JMV conceived and designed the research. STS and JL acquired the data and wrote the initial draft. STS, JER, FD, JME and JL analysed and interpreted the data. STS, FD and JMV handled the distribution of resources and supervision. STS and JL performed the literature review. JMV, JER, FD, JL and STS made critical revisions of the manuscript. All authors read and approved the final manuscript.
